# Altered lipid profile and reduced neuronal support in human induced pluripotent stem cell‐derived astrocytes from adrenoleukodystrophy patients

**DOI:** 10.1002/jimd.12832

**Published:** 2024-12-20

**Authors:** Roberto Montoro Ferrer, Yorrick R. J. Jaspers, Inge M. E. Dijkstra, Nicole Breeuwsma, Jan‐Bert van Klinken, Cato Romero, Marc Engelen, Stephan Kemp, Vivi M. Heine

**Affiliations:** ^1^ Laboratory Genetic Metabolic Diseases, Department of Laboratory Medicine, Amsterdam UMC, Amsterdam Gastroenterology Endocrinology Metabolism University of Amsterdam Amsterdam The Netherlands; ^2^ Department of Pediatric Neurology Emma Children's Hospital, Amsterdam UMC, Amsterdam Leukodystrophy Center, Amsterdam Neuroscience, University of Amsterdam Amsterdam The Netherlands; ^3^ Department of Complex Trait Genetics Centre for Neurogenomics and Cognitive Research, Amsterdam Neuroscience, Vrije Universiteit Amsterdam Amsterdam The Netherlands; ^4^ Department of Child and Adolescence Psychiatry Emma Children's Hospital, Amsterdam UMC Location, Vrije Universiteit Amsterdam, Amsterdam Neuroscience Amsterdam The Netherlands; ^5^ Core Facility Metabolomics Amsterdam UMC Location, University of Amsterdam Amsterdam The Netherlands

**Keywords:** *ABCD1* gene, astrocytes, human‐induced pluripotent stem cells, lipid homeostasis, peroxisome, X‐linked adrenoleukodystrophy

## Abstract

X‐linked adrenoleukodystrophy (ALD) is a peroxisomal disorder resulting from pathogenic variants in the *ABCD1* gene that primarily affects the nervous system and is characterized by progressive axonal degeneration in the spinal cord and peripheral nerves and leukodystrophy. Dysfunction of peroxisomal very long‐chain fatty acid (VLCFA) degradation has been implicated in ALD pathology, but the impact on astrocytes, which critically support neuronal function, remains poorly understood. Fibroblasts from four ALD patients were reprogrammed to generate human‐induced pluripotent stem cells (hiPSC). hiPSC‐derived astrocytes were generated to study the impact of ALD on astrocytic fatty acid homeostasis. Our study reveals significant changes in the lipidome of ALD hiPSC‐derived astrocytes, characterized by an enrichment of VLCFAs across multiple lipid classes, including triacylglycerols, cholesteryl esters, and phosphatidylcholines. Importantly, ALD hiPSC‐derived astrocytes not only exhibit intrinsic lipid dysregulation but also affect the dendritic tree complexity of neurons in co‐culture systems. These findings highlight the cell‐autonomous effects of pathogenic variants in the ABCD1 protein on astrocytes and their microenvironment, shed light on potential mechanisms underlying ALD neuropathology, and underscore the critical role of astrocytes in neuronal health.

## 1 INTRODUCTION

X‐linked adrenoleukodystrophy (ALD) (OMIM # 300100) is the most common peroxisomal disorder caused by pathogenic variants in *ABCD1*,^1^, ^2^ which encodes a peroxisomal membrane transporter and affects the brain, spinal cord, adrenal glands, and testes.^3^, ^4^ Slowly progressive axonal degeneration in the spinal cord (specifically the dorsal columns and corticospinal tracts) and peripheral nerves (“adrenomyeloneuropathy [AMN]”) is the core disease that presents in adulthood in both male and female patients, and there is currently no disease‐modifying treatment.^3^, ^5^ Additionally, male patients may develop leukodystrophy (“cerebral ALD”), characterized by progressive degeneration of the cerebral white matter in a highly characteristic pattern in which axonal loss appears to precede myelin destruction. Although leukodystrophy is primarily seen in males, isolated cases of female patients with cerebral ALD have been reported.^6^, ^7^ The leukodystrophy can occur at any age, with a peak incidence between 4 and 8 years of age.^3^ Magnetic resonance imaging (MRI) abnormalities appear long before symptoms of neurocognitive decline and motor deficits, providing a therapeutic window for allogeneic hematopoietic stem cell transplantation (HSCT) that can halt further progression of the leukodystrophy.^8^ Biochemically, peroxisomal dysfunction in ALD is characterized by elevated levels of very long‐chain fatty acids (VLCFA).^9^ However, the precise mechanisms by which deficits in VLCFA degradation may lead to demyelination and/or axonal degeneration remain poorly understood.

Peroxisomes are prominently distributed in various cell types of the central nervous system, particularly within glial cells.^10^ One of their key roles in this system is the catabolism of saturated VLCFAs, particularly C24:0 and C26:0, as the initial steps of VLCFA degradation take place exclusively in peroxisomes.^11^ VLCFA breakdown involves multiple cycles, each resulting in the formation of shortened fatty acid chains that are subsequently enzymatically activated into acyl‐CoA derivatives. These acyl‐CoA molecules serve dual purposes: they can either be esterified into membrane lipids, particularly plasmalogens, contributing to cholesterol and phospholipid synthesis,^12^, ^13^ or they can be degraded via mitochondrial β‐oxidation to provide cellular energy,^14^ primarily in glial cells. In particular, the relatively slower rate of β‐oxidation in neurons has been postulated to confer protection against oxidative stress and neuronal hypoxia.^15^ However, fatty acid oxidation plays a critical role in oligodendrocytes, facilitating lipid turnover and providing essential components for the synthesis of new myelin lipids.^16^, ^17^ Moreover, under glucose deprivation, oligodendrocytes support axonal function by metabolizing myelin lipids through β‐oxidation.^18^ Although astrocytes have high levels of ABCD1 protein and peroxisomal β‐oxidation plays an important role in brain energy supply, few studies have focused on astrocytic defects in ALD.

Astrocytes play a central role in maintaining neuronal function through multiple metabolic processes, encompassing nutrient provision, lipid metabolism, neurotransmitter regulation, and oxidative stress management.^19^ The energetic demands of these functions require robust mitochondrial activity, with fatty acid oxidation emerging as the primary supplier of acetyl‐CoA for the tricarboxylic acid (TCA) cycle in astrocytes.^20^ Excess fatty acids generated by overactive neurons are sequestered by astrocytes via ApoE‐containing lipid particles and stored in lipid droplets after esterification.^21^ The efficient catabolic machinery within astrocytes is essential for processing these lipids and maintaining neuronal support. Neurons exposed to astrocytes deficient in fatty acid degradation exhibit impaired glycolytic and mitochondrial metabolism, reduced synaptic density, and increased susceptibility to neuroinflammation and neurodegeneration.^22^ Neurons have a limited capacity to degrade fatty acids, and the accumulation of excess fatty acids can lead to increased levels of reactive oxygen species (ROS) and lipid peroxidation.^21^, ^23^ Furthermore, impaired lipid biogenesis in astrocytes adversely affects synapse development and myelination.^24^, ^25^ Thus, the intricate interplay of lipid metabolism between neurons and astrocytes serves as a critical protective mechanism against ROS and fatty acid‐induced toxicity, preserving neuronal integrity and function. Whether ALD astrocytes are primarily affected by ABCD1 deficiency and whether impaired astrocytic peroxisomal VLCFA degradation could trigger neuronal pathology remains to be shown.

In this study, we investigate the impact of ABCD1 deficiency on fatty acid homeostasis in astrocytes using human‐induced pluripotent stem cell (hiPSC) technology. Our results show significant alterations in the lipidome of ALD hiPSC‐derived astrocytes, with notable changes in triacylglycerols (TG), cholesteryl esters (CE), and phosphatidylcholines (PC). Notably, ALD hiPSC‐derived astrocytes not only exhibit intrinsic lipid dysregulation, but also demonstrate impaired support for neurons in co‐culture systems. These results highlight the profound, cell‐autonomous effects of the ALD genetic background on hiPSC‐derived astrocytes and their microenvironment.

## 2 METHODS

### 2.1 Generation of hiPSCs

Fibroblasts from four ALD patients were reprogrammed with a lentiviral construct overexpressing OCT4, SOX2, KLF‐4, and C‐MYC, and fibroblasts from 1 anonymous donor were reprogrammed using the CytoTune™‐iPSC 2.0 Sendai Reprogramming Kit, containing Oct3/4, Sox2, Klf‐4, and c‐Myc. Generated hiPSC lines were confirmed for pluripotency by immunocytochemistry, polymerase chain reaction (PCR), alkaline phosphatase, embryoid body formation assay, karyotyping, and/or a Pluritest. Control hiPSC were earlier generated and characterized for pluripotency.^26^ See Table S1 for an overview of the hiPSC lines. Control and ALD hiPSCs were maintained on Vitronectin XF (Stem Cell Technologies, Vancouver, BC, Canada, #07180)‐coated plates in TeSR™‐E8™ medium (Stem Cell Technologies, Vancouver, BC, Canada, #05990). Medium was refreshed daily, and cells were passaged once a week using Gentle Cell Dissociation Reagent (Stem Cell Technologies, #100–0485) according to the manufacturer's protocol. Cells were split in 1:10 to 1:50 ratios to a new well for further maintenance.

### 2.2 Generation of hiPSC‐derived astrocytes

hiPSC‐derived astrocytes were generated according to previously established protocols with minimum modifications.^26^, ^27^ Briefly, hiPSCs colonies were enzymatically detached with ethylenediaminetetraacetic acid (EDTA) and transferred into low‐attachment plates for the formation of embryoid bodies (EBs) in Neural Maintenance Medium (NMM) consisting of 1:1 Dulbecco's MEM/F12 (DMEM/F12) medium and Neurobasal Medium supplemented with 0.5% N2, 1% B27, 5 μg/mL human insulin, 1.5 mM L‐glutamine, 100 μM non‐essential amino acids (NEEA), 10 μM β‐mercaptoethanol, and 100 U/mL penicillin–streptomycin. On Day 0, NMM was supplemented with 20 ng/mL epidermal growth factor (EGF), 4 ng/mL fibroblast growth factor 2 (FGF2), 40 ng/mL triiodothyronine (T3), and 10 μM Rho‐associated coiled‐coil containing protein kinase (ROCK) inhibitor. From Day 2, the medium was additionally supplemented with 10 μM retinoic acid (RA). On Day 10, EBs were plated on Geltrex‐coated plates in NMM containing 20 ng/mL EGF and 40 ng/mL T3. If the center of the EBs darkened before Day 10, EBs were plated earlier, but not before Day 4. However, the medium composition was not changed until Day 10. From this moment, cells were dissociated 1:2/1:3 with Accutase at 37°C for 5–10 min. On Day 18, the medium was changed to NMM with B‐27 vitamin A supplemented with 20 ng/mL EGF and 40 ng/mL T3. On Day 37, the medium was changed to NMM with B27 vitamin A supplemented with 5 ng/mL EGF, 40 ng/mL T3, 5 ng/mL FGF2, 1 μg/mL laminin, and 50 μg/mL vitamin C. On Day 39, 0.1 μM dorsomorphin (DM) was additionally supplemented. On Day 42, EGF and FGF2 were removed from the supplements added. From Day 45 onwards, NMM medium was replaced with ScienCell Astrocyte Medium to promote astrocytic differentiation. Two‐thirds of the medium was changed every other day for the first 45 days, and one‐half of the medium was changed every other day for the rest of the protocol. To cryo‐preserve hiPSC‐derived astrocytes, cells were resuspended in Astrocyte Medium +10% dimethyl sulfoxide (DMSO), transferred into Mr. Frosty freezing container (Thermo Scientific 5100‐0001) in cryogenic vials, and then transferred to liquid nitrogen for long storage.

### 2.3 Generation of hiPSC‐derived motor neurons

hiPSC‐derived motor neurons were generated following Du et al.^28^ with the following changes. Briefly, hiPSC colonies were dissociated with Accutase at 37°C for 5 min., made into a single‐cell suspension, and 9000 cells were plated per well into ultra‐low attachment U‐bottom 96‐well plates (Corning, #CLS7007) in 150 μL of TeSR™‐E8™ medium (Stem Cell Technologies, #5940) supplemented with 4 ng/mL FGF2 and 10 μM ROCK inhibitor. On Day 2, 150 μL of NMM supplemented with 3 μM CHIR99021 (CHIR), 2 μM SB‐431542, 0.2 μM DM, and 0.1 mM vitamin C was added (motor neuron progenitor medium 1 [MNP1]). On Day 8, MNP1 was changed to MNP2, consisting of NMM supplemented with 1 μM CHIR, 2 μM SB‐431542, 0.2 μM DM, 0.1 μM RA, 0.25 μM smoothened agonist (SAG), and 0.1 mM vitamin C. Two‐thirds of the medium was changed daily from Day 2 to Day 7, and every other day from Day 8 onwards. On Day 14, patterned EBs were dissociated into single cells with Accutase at 37°C for 15 min in rotation and plated in geltrex‐coated plates at a cell density of 70 000 cells/cm^2^ in MNP1 (supplemented with 10 μM ROCK inhibitor on the plating day). On Day 22, patterned progenitors were dissociated into single cells with Accutase at 37°C for 5 min and plated on poly‐L‐ornithine and laminin‐coated glass coverslips at a cell density of 35 000 cells/cm^2^ in motor neuron differentiation (MN) medium consisting of NMM supplemented with 0.5 μM RA, 0.1 μM SAG, 0.1 mM vitamin C, 1 μM cyclyc AMP (cAMP), 20 ng/mL brain‐derived neurotrophic factor (BDNF), 10 ng/mL neurotrophin 3 (NT3), 10 ng/mL glial cell line‐derived neurotrophic factor (GDNF), 10 ng/mL insulin growth factor 1 (IGF‐1), and 10 μM (2S)‐N‐[(3,5‐difluorophenyl)acetyl]‐L‐alanyl‐2‐phenylglycine 1,1‐dimethyl ester (DAPT) (10 μM ROCK inhibitor was added on the plating day). From Day 22 onwards, half of the medium was changed every 3–4 days for the rest of the protocol.

### 2.4 Astrocyte‐motor neuron co‐cultures

To generate astrocyte‐motor neuron co‐cultures, Day 22 motor neuron progenitors were dissociated and plated at a cell density of 35 000 cells/cm^2^ on top of a monolayer of Day 90 hiPSC‐derived astrocytes in MN medium (supplemented with 10 μM ROCK inhibitor on the plating day). After 7 days in co‐culture, 2 μM AraC was added once to prevent astrocyte proliferation. Half of the medium was changed every 3–4 days.

### 2.5 Lipidomics

Lipidomics analysis was performed as previously described.^6^ In a 2 mL tube, 200 μg was added and mixed with a mix of internal standards for different lipid classes, including 0.1 nmol cardiolipin (CL(14:0/14:0/14:0/14:0)), 2.0 nmol (PC(14:0/14:0)), 0.1 nmol phosphatidylglycerol (PG(14:0/14:0)), 5.0 nmol phosphatidylserine (PS(14:0/14:0)), 0.5 nmol phosphatidylethanolamine (PE(14:0/14:0)), 0.5 nmol phosphatidic acid (PA(14:0/14:0)), 2.125 nmol sphingomyelin (SM(d18:1/12:0)), 0.02 nmol lysophosphatidylglycerol (LPG(14:0)), 0.1 nmol lysophosphatidylethanolamine (LPE(14:0)), 0.5 nmol lysophosphatidylcholine (LPC(14:0)), 0.1 nmol lysophosphatidic acid (LPA(14:0)), 0.5 nmol phosphatidylinositol (PI(8:0/8:0)), 0.5 nmol diglycerides (DG(14:0/14:0)), 0.5 nmol triglycerides (TG(14:0/14:0/14:0)), 2.5 nmol CE (D_7_‐CE(16:0)), 0.125 nmol of sphingosine and ceramide mix (Avanti Polar Lipids) dissolved in 1:1 (v/v) methanol:chloroform. Next, 1.5 mL of 1:1 (v/v) methanol: chloroform was added to each sample. The mixture was sonicated in a water bath (5 min) and centrifuged (4°C, [16 000 × g, 10 min]). The supernatant was transferred to a 1.5 mL glass autosampler vial and evaporated under a stream of nitrogen at 45°C. The dried lipids were reconstituted in 100 μL of 1:1 (v/v) chloroform:methanol. Chromatographic separation of lipids was done using a Thermo Fisher Scientific Ultimate 3000 binary ultra‐performance liquid chromatography (UPLC) using a normal phase and a reverse phase column in separate runs. Normal‐phase separation was done using a Phenomenex® LUNA silica, 250 × 2 mm, 5 μm, 100 Å column. Column temperature was held constant at 25°C. The composition of the mobile phase A consisted of 85:15 (v/v) methanol:water containing 0.0125% formic acid and 3.35 mmol/L ammonia, and the composition of the mobile phase B consisted of 97:3 (v/v) chloroform:methanol containing 0.0125% formic acid. The liquid chromatography (LC) gradient started at 10% A for 0–1 min, 20% A at 4 min, 85% A at 12 min, 100% A at 12.1 min, 100% A for 12.1–14 min, 10% A at 14.1 min, 10% A for 14.1–15 min using a flow rate of 0.3 mL/min. Reversed‐phase separation was done using a Waters HSS T3 column (150 × 2.1 mm, 1.8 μm particle size). The composition of the mobile phase A consisted of 4:6 (v/v) methanol:water and B 1:9 (v/v) methanol: isopropanol, both containing 0.1% formic acid and 10 mmol/L ammonia. The gradient started at 100% A, going to 80% A at 1 min and 0% A at 16 min, 0% A for 16–20 min, 100% A at 20.1 min, and 100% A for 20.1–21 min. The column temperature was held constant at 60°C, and a flow rate of 0.4 mL/min was used. After LC separation, lipids were detected using a Q Exactive Plus Orbitrap mass spectrometer (Thermo Scientific) using negative and positive ionization. The spray voltage was 2500 V and nitrogen was used as the nebulizing gas. A resolution of 280 000 was used in a mass range of *m*/*z* 150 to *m*/*z* 2000.

### 2.6 Metabolomics

Metabolomics was performed as previously described,^29^ with minor adjustments. In a 2 mL tube, the following amounts of internal standard dissolved in water were added to each sample of 200 μg: adenosine‐^15^N_5_‐monophosphate (5 nmol), adenosine‐^15^N_5_‐triphosphate (5 nmol), D_4_‐alanine (0.5 nmol), D_7_‐arginine (0.5 nmol), D_3_‐aspartic acid (0.5 nmol), D_3_‐carnitine (0.5 nmol), D_4_‐citric acid (0.5 nmol), ^13^C_1_‐citrulline (0.5 nmol), ^13^C_6_‐fructose‐1,6‐diphosphate (1 nmol), ^13^C_2_‐glycine (5 nmol), guanosine‐^15^N_5_‐monophosphate (5 nmol), guanosine‐^15^N_5_‐triphosphate (5 nmol), ^13^C_6_‐glucose (10 nmol), ^13^C_6_‐glucose‐6‐phosphate (1 nmol), D_3_‐glutamic acid (0.5 nmol), D_5_‐glutamine (0.5 nmol), D_5_‐glutathione (1 nmol), ^13^C_6_‐isoleucine (0.5 nmol), D_3_‐lactic acid (1 nmol), D_3_‐leucine (0.5 nmol), D_4_‐lysine (0.5 nmol), D_3_‐methionine (0.5 nmol), D_6_‐ornithine (0.5 nmol), D_5_‐phenylalanine (0.5 nmol), D_7_‐proline (0.5 nmol), ^13^C_3_‐pyruvate (0.5 nmol), D_3_‐serine (0.5 nmol), D_6_‐succinic acid (0.5 nmol), D_4_‐thymine (1 nmol), D_5_‐tryptophan (0.5 nmol), D_4_‐tyrosine (0.5 nmol), D_8_‐valine (0.5 nmol). Solvents were then added to give a total volume of 500 μL methanol and 500 μL water. A 5 mm stainless steel bead was added, and a Qiagen TissueLyser II was used for 5 min at 30 times/s to homogenize each sample before adding 1 mL of chloroform. After thorough mixing, the samples were centrifuged at 14 000 rpm for 10 min. The polar top layer was transferred to a new 1.5 mL tube and dried at 60°C using a vacuum concentrator. Dried samples were reconstituted in 100 μL of 6:4 (v/v) methanol:water. Metabolites were analyzed using a Waters Acquity ultra‐high performance liquid chromatography (HPLC) system coupled to a Bruker Impact II™ Ultra‐High Resolution Qq‐Time‐Of‐Flight mass spectrometer. Samples were kept at 12°C during analysis, and 5 μL of each sample was injected. Chromatographic separation was performed on a Merck Millipore SeQuant ZIC‐cHILIC column (PEEK 100 × 2.1 mm, 3 μm particle size). The column temperature was maintained at 30°C. The mobile phase consisted of (A) 1:9 (v/v) acetonitrile:water and (B) 9:1 (v/v) acetonitrile:water, both containing 5 mmol/L ammonium acetate. Using a flow rate of 0.25 mL/min, the LC gradient consisted of: Dwell at 100% solvent B, 0–2 min; ramp to 54% solvent B at 13.5 min; ramp to 0% solvent B at 13.51 min; dwell at 0% solvent B, 13.51–19 min; ramp to 100% solvent B at 19.01 min; dwell at 100% solvent B, 19.01–19.5 min. The column was equilibrated by increasing the flow rate to 0.4 mL/min at 100% B for 19.5–21 min. MS data were acquired using negative and positive ionization in full scan mode over the range of *m*/*z* 50–1200. Data were analyzed using Bruker TASQ software version 2021.1.2.452. All reported metabolite intensities were normalized to the sum of all adenosine nucleotides and to internal standards with comparable retention times and MS response. Metabolite identification was based on a combination of accurate masses, (relative) retention times, ion mobility data, and fragmentation spectra compared to analysis of a library of standards.

### 2.7 LPC(26:0) quantification

Analysis of LPC(26:0) was performed as described earlier.^30^ Briefly, 100 μg of protein was extracted with 10 μL of an internal standard solution containing 1 μmol/L D_4_‐C26:0‐lysoPC in 0.5 mL of acetonitrile by ultrasonication for 5 min in a sonicator bath (Branson 3510) at room temperature. After centrifugation (5 min, 14 000 RPM), the resulting acetonitrile layer was transferred to a new glass tube and evaporated under a constant stream of nitrogen at 40°C. The samples were then reconstituted in 50 μL methanol, transferred to a sample vial, and capped. High‐perfomance liquid chromatography tandem mass spectrometry (HPLC–MS/MS) analysis samples were injected using an ACQUITY UPLC system (Waters, Milford, MA, USA) on a 50 × 2.1 μm, 2.6 μm particle diameter Kinetex C8 column (Phenomenex, Torrance, CA, USA). The column was held at a constant temperature of 50°C. The composition of mobile phase A was 0.1% formic acid in water, and mobile phase B was 0.1% formic acid in methanol. The gradient used was as follows: *T* = 0 min: 36% A, 64% B, flow 0.4 mL/min toward *T* = 6 min: 0% A, 100% B, flow 0.4 mL/min; *T* = 6–11 min: 0% A, 100% B, flow 0.4 mL/min, and *T* = 11–11.1 back to 36% A, 64% B, flow 0.4 mL/min. Detection was done using a Quattro Premier XE (Waters, Milford, MA, USA) using electrospray ionization in positive mode. The source temperature was 130°C, and capillary voltage was 3.5 kV. Multiple reaction monitoring (MRM) was done on masses 636.50 > 104.10 and 640.50 > 104.10 with a dwell time of 0.03 s. Argon was used as a collision gas.

### 2.8 Fatty acid treatment

VLCFA β‐oxidation of D_3_C22:0 (30 μM; CDN isotopes, #D‐5708) and elongation of LCFA D_3_C16:0 (100 μM; CDN isotopes, #D‐1655) were measured as previously described.^31^ Astrocytes plated in six‐well plates were incubated with the stable isotope‐labeled fatty acids for 72 h. For treatment experiments, astrocytes plated in six‐well plates (300 k cells/well) or in 96‐well plates (9 k cells/well) were treated with 10 μM C26:0, C26:1, or vehicle (DMSO, 0.17%) for 24 h. Before the fatty acids were added to the tissue culture medium, the stock solution was heated to 70°C in a heat block and immediately diluted in tissue culture medium (heated to 37°C). The final DMSO concentration was kept under 1%. Equivalent DMSO concentration was used as a vehicle control. Fatty acids were added on Day 1 and not refreshed until the end of the experiment.

### 2.9 Immunostaining

Cells were washed with phosphate‐buffered saline (PBS) and fixed with 4% paraformaldehyde (PFA, diluted from a 16% solution; ProSciTech, C004) for 15 min at room temperature. After 6× PBS washes of 5 min, cells were blocked for 1 h at room temperature with PBS + 5% goat serum (Thermo Scientific, Cat# 16210) + 0.1% bovine serum albumine (BSA) (Merck, A9418) + 0.3% Triton X‐100 (Merck, T8787). Primary antibodies in blocking buffer were incubated for 1 h at room temperature and overnight at 4°C. Antibodies used were glial fibrillary acidic protein (GFAP) (Merck, #G3893, dilution 1:1000), S100β (ProteinTech, 15 146‐1‐AP, dilution 1:1000), NESTIN (BD Bioscience, #611658, dilution 1:500), and ID3 (Cell Signaling, #9837, dilution 1:250), CD44 (DSHB, H4C4, dilution 1:100), SOX9 (Cell Signaling, #82630, dilution 1:500), MAP2 (Merck, AB5543, dilution 1:2500), synaptophysin (SYN) (Synaptic Systems, #101011, dilution 1:500), ABCD1 (Abcam, ab197013, dilution 1:500). The next day, after 6× PBS washes of 5 min, cells were incubated with secondary antibodies (Thermo Scientific, Alexa Fluor‐488, 594, and 647; dilution 1:1000) for 2 h at room temperature. Afterwards, cells were washed 6× 5 min with PBS, incubated with diamidino‐2‐phenylindole (DAPI, Merck, D9542, dilution 1:1000) for 2 min at room temperature, washed once with PBS and embedded with Fluoromount‐G (Southern Biotech, #0100‐01).

### 2.10 Sudan III and F‐actin staining

hiPSC‐derived were astrocytes plated in 96‐well plates (9 k cells/well) and cultured for 96 h were fixed with 4% PFA for 15 min at room temperature. After 6× PBS washes of 5 min, cells were incubated with Alexa Fluor 488 phalloidin (Thermo Scientific, R37110) 1 h at room temperature (1 drop/mL) and then washed three times with PBS. Cells were then incubated with 60% isopropanol for 5 min at room temperature. Afterwards, cells were incubated with Sudan III solution (Morphisto, #10396; 10 mL of Sudan III Solution diluted in 1.5 mL of Mili‐Q water and 0.2 μm filtered syringe) for 20 min at room temperature. Cells were washed with Mili‐Q, and plates were taken to image on a Cell Insight CX7 HCS platform (Thermo Scientific, cat# N01002INF).

### 2.11 Cell Insight CX7 HCS platform image analysis

High‐content microscopy was performed on a CellIInsight CX7 HCS platform (Thermo Fisher) in widefield mode with a 20× objective. Z‐stacks were acquired with a step size of 0.6 μm over a total depth of 6.6 μm of hiPSC‐derived astrocytes stained for lipid droplets (Sudan III), ABCD1, and GFAP. To quantify lipid droplets in untreated control and ALD hiPSC‐derived astrocytes, two wells per independent differentiation batch (*n* = 3) from 96‐well plates were imaged. For treated control hiPSC‐derived astrocytes, three wells per independent batch (*n* = 3) from 96‐well plates were imaged. A total of 12 fields per well were imaged. Maximum intensity projections were analyzed using Columbus analysis software (PerkinElmer v2.5.2.124862) with custom, in‐house developed scripts. Briefly, cell number quantification was based on DAPI fluorescence, with nuclei touching the image borders excluded from analysis. Following DAPI‐based cell detection, cytoplasms were defined by F‐actin fluorescence and lipid droplet puncta were quantified within these regions. ABCD1 puncta were quantified and normalized to the total number of DAPI nuclei to estimate the average ABCD1 puncta per cell. To determine the percentage of GFAP+ astrocytes, cells were first identified by DAPI fluorescence, and GFAP positivity was assessed based on fluorescence intensity thresholds (presence or absence of fluorescence).

### 2.12 Neuronal analysis

Confocal images were acquired with Nikon ECLIPSE Ti inverted microscope (Nikon Corporation) controlled by NIS‐Elements 4.30 software (Nikon Corporation). For synapse density and dendritic tree reconstruction, images were taken using a 40× oil objective (NA = 1.3) with Z‐stacks of μm at a 0.6 μm step size. SYN puncta and Sholl analysis were analyzed using SynJ and Simple Neurite Tracer (SNT) Neuroanatomy plugins for Fiji, respectively.^32^, ^33^ For SYN puncta quantification, somas, neurites, and SYN puncta were automatically detected and analyzed using the SynJ plugin. Dendrites were defined by MAP2 positivity, with parameters set to an estimated line width of 9 pixels and a minimum neurite length of 40 pixels to create a dendritic mask. SYN puncta within this mask were then automatically quantified using the SynJ plugin in ImageJ, with the following detection parameters: tophat radius = 2 pixels to enhance contrast, synapse radius = 2 pixels to set particle size, synapse smooth = 0.5 to refine edges, and synapse threshold = 1 standard deviation above mean neurite intensity to identify positive puncta. For Sholl analysis, neurons were reconstructed using SNT semi‐automatically tracing, with paths being blindly reconstructed in a maximum projection image using in parallel the corresponding Z‐stack to precisely reconstruct the dendritic tree. To assess differences in dendritic arborization, we applied a linear mixed model in R using the package “ *nlme* ” (v. 3.1‐164). Before analyses could be performed, we filtered out neurons with outlying number of intersections. Within ALD and control condition neurons separately, we aggregated the total number of intersections for each individual neuron and removed those that were considered outliers according to the interquartile range (IQR) method (i.e., remove *value* if <Q1–1.5 × IQR or remove *value* if >Q3 + 1.5 × IQR). This resulted in 10 neurons being removed from the control condition and 11 neurons being removed from the ALD condition. Number of intersections was then log‐transformed (Log^2^(*Intersection* + 1)) to improve the normal distribution of the residuals.

To account for the multi‐level dependency within the data (that is, multiple observations of intersections stemming from the same neuron, and multiple neurons stemming from the same coverslip and patient/control subject), we defined a random effects model. This model allows for a varying intercept at each level of the data, thus accounting for any variation that would be due to, for example, genetic background (from the subjects) and technical variation (from the coverslips and neurons). The random effects model was compared to a fixed effects model using a likelihood ratio Chi^2^ test to assess the degree of dependency in the data. The fit of the random effects model was significantly better than the fixed effects model (Akaike Information Criterion (AIC) AIC_random_ = 26 866, AIC_fixed_ = 35 447; Chi^2^(3) = 8586.82, one‐tailed test *p* < 5.0 × 10^−5^), with a substantial intra‐cluster correlation coefficient (ICC) of 0.398. This indicates that observations stemming from the same neuron from the same coverslip and subjects are correlated and must be corrected for to prevent type‐1 error inflation. Removing any level of dependency (neuron, coverslip, or subject) resulted in significantly poorer fit compared to the full random effects model (one‐tailed test *p* < 0.0001); hence, the full random effects model was used in the main analysis.

To test the overall difference in mean intersections between neurons from the ALD and control condition, the condition (ALD or control) was set as a predictor of observed intersections. The variance of the random effects was used to calculate the *unexplained* ICC, which was 0.296. Thus, the *explained* ICC by including the ALD/control condition, the model was 0.398–0.296 = 0.102, meaning that the ALD/control condition explains 10.2% of the variance in observed intersections. Residuals of the model showed little sign of violating the assumption of normality and homoscedasticity. The same model was repeated in stratified analyses for intersections observed within radii 20–29 and 80–89 μm. All statistical tests were, if not stated otherwise, two‐tailed and tested against *α* < 0.05.

### 2.13 RNA isolation and cDNA generation

RNA was isolated from cells by incubation in 750 μL TRIzol (Thermo Scientific, #15596018). Per 750 μL TRIzol, 150 μL chloroform was added, and samples were shaken vigorously and left to incubate for 2–3 min at room temperature. Samples were centrifuged at 13 000 g for 10 min at 4°C. The upper aqueous phase was transferred to a new tube, and 350 μL isopropanol was added. After 10–15 min incubation at room temperature, samples were centrifuged at 13 000 g for 10 min at 4°C. The supernatant was removed by decanting, and the pellet was washed with 1 mL of 70% ethanol. Remaining ethanol was removed with a pipette and the pellet was air dried for 5 min. Samples were dissolved in 10–30 μL DMPC‐H_2_O, and RNA concentration and 260/280 ratios were measured with a NanoDrop spectrophotometer (NanoDrop 2000 Thermo Scientific). cDNA was made from 1 μg RNA using Superscript IV (Invitrogen, #18090010) and 50 ng/μL random hexamer primers (Qiagen, #79236) according to the manufacturer's protocol.

### 2.14 Quantitative PCR

For quantitative PCR (qPCR) analysis, cDNA samples were diluted tenfold and run on a Lightcycler 480 (Roche) using a Sensifast SYBR Hi‐ROX‐kit (Bioline, #BIO‐92020) according to the manufacturer's protocol. All samples were run in duplicate or triplicate. An overview of the primers used can be found in Table S2. The ∆∆Ct values were calculated by correcting for the housekeeping gene. The ∆∆Ct values were converted to fold changes in gene expression by 2^−∆∆Ct^. The fold changes were averaged between samples of the same line and differentiation batch, and these averages were used for statistical analysis.

### 2.15 Quantification and statistical analysis

Data are presented as mean ± standard deviation (SD) unless otherwise noted. The distribution of raw data was tested for normality. Details of the statistical test used for each experiment are provided in the figure legends and in the Results section. In general, *n* values refer to the number of individual donor lines, and details are provided for each experiment in the figure legends. Sholl analysis was performed in a blinded fashion.

## 3 RESULTS

### 3.1 ALD hiPSC‐derived astrocytes present deficits in VLCFA metabolism

**FIGURE 1:**
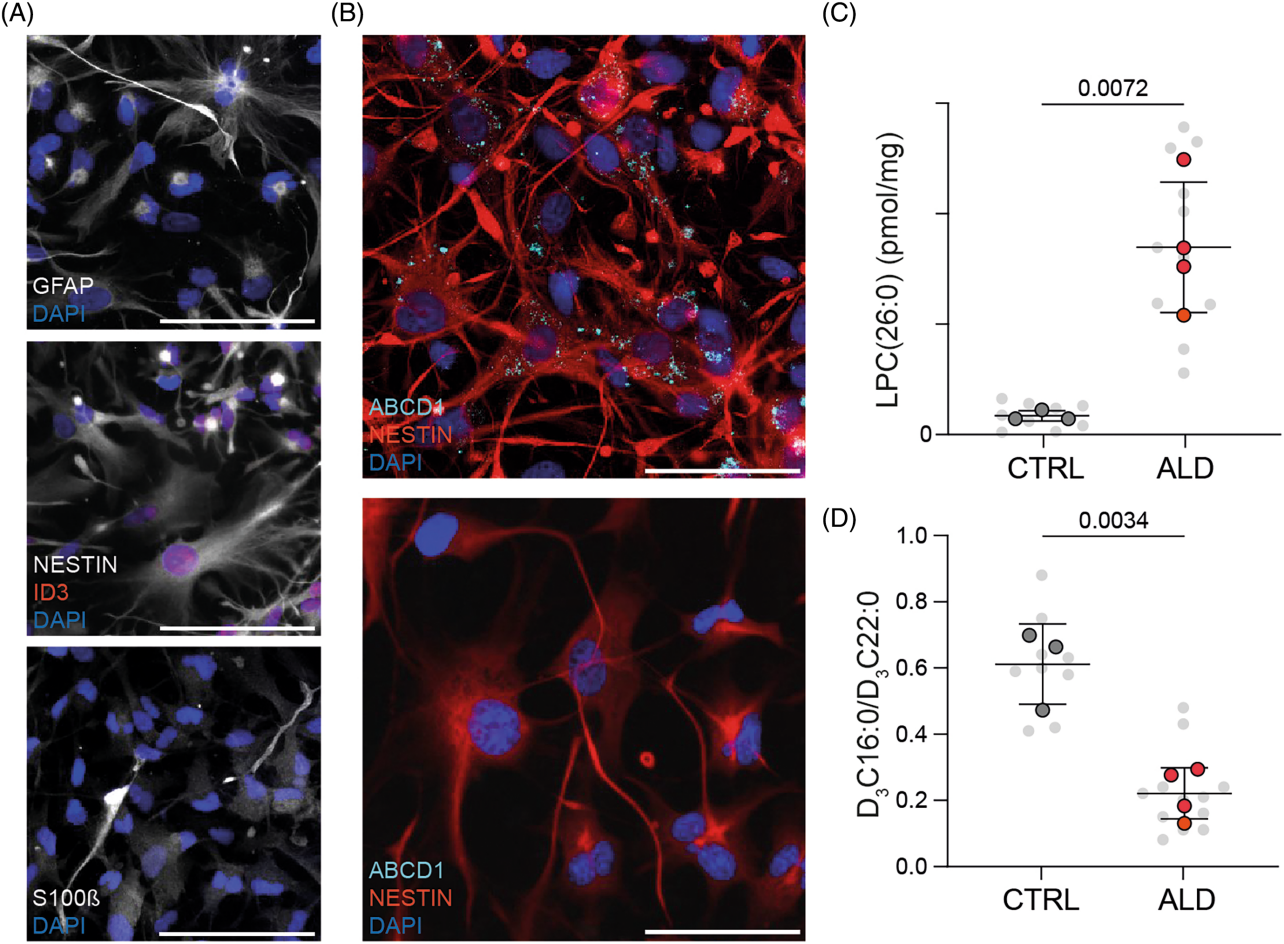
Adrenoleukodystrophy (ALD) human‐induced pluripotent stem cells (hiPSC)‐derived astrocytes present deficits in very long‐chain fatty acid metabolism. (A) Immunofluorescence images of control and ALD hiPSC‐derived astrocytes positive for astrocyte‐associated markers GFAP, ID3, NESTIN, and S100β at Day ~60 (scale bar, 100 μm). (B) Confocal immunofluorescence image of control hiPSC‐derived astrocytes positive for CD44 and ABCD1 at Day ~60 (scale bar, 100 μm). (C) C26:0‐lysophosphatydylcholine (LPC(26:0)) levels in control (*n* = 3) and ALD (*n* = 4) hiPSC‐derived astrocytes at Day 70; three independent batches per line; mean ± SD. (D) Control (*n* = 3) and ALD (*n* = 4) hiPSC‐derived astrocytes were cultured with 30 μM D_3_‐C22:0 for 3 days. Peroxisomal VLCFA β‐oxidation is expressed as the ratio of D_3_‐C16:0 (product) to D_3_‐C22:0 (substrate); three independent batches per line; mean ± SD. In (C) and (D), the mean values of each donor are shown in color and individual data points of each differentiation batch (*n* = 3) are shown in gray in the background. *p* values were calculated by two‐tailed nested *t*‐test with all individual data points nested per group (control *n* = 3 and ALD *n* = 4) and are shown as numbers when significant. DAPI, diamidino‐2‐phenylindole.

We selected four ALD patients, of whom three were diagnosed with cerebral ALD and one with spinal cord disease (Table S1). Patient fibroblasts were reprogrammed into hiPSC and characterized for pluripotency using standard assays (Figure S1). We then generated hiPSC‐derived astrocytes from pre‐existing fully characterized hiPSC from healthy individuals and ALD lines using a previously established protocol.^27^, ^34^ At Day 60 of differentiation, hiPSC‐derived astrocytes from all control and ALD lines were examined for typical astrocytic morphology and expression of the astrocyte‐associated markers GFAP, S100β, NESTIN, ID3, SOX9, and CD44 (Figures 1A and S2). No obvious deficits were observed in the ALD astrocytes. To confirm whether hiPSC‐derived astrocytes in culture express ABCD1, we performed immunocytochemical labeling of ABCD1 in control hiPSC‐derived astrocytes (Figure 1B).

Using liquid chromatography tandem mass spectrometry (LC–MS/MS), we confirmed that 70‐day‐old ALD hiPSC‐derived astrocytes had elevated levels of C26:0‐lysophosphatydylcholine (LPC(26:0)) (Figure 1C), an indication of VLCFA accumulation. On average, LPC(26:0) levels in ALD hiPSC‐derived astrocytes were nearly 10 times higher than in control hiPSC‐derived astrocytes.

To investigate whether ALD astrocytes have impaired peroxisomal VLCFA β‐oxidation capacity, we incubated hiPSC‐derived astrocytes with the stable isotope‐labeled docosanoic acid (D_3_C22:0). ALD hiPSC‐derived astrocytes exhibited a lower D_3_C16:0/D_3_C22:0 ratio, indicating significantly reduced peroxisomal VLCFA β‐oxidation capacity (Figure 1D). These findings highlight a deficiency in VLCFA metabolism in ALD hiPSC‐derived astrocytes and demonstrate that ALD hiPSCs can differentiate into astrocytes and exhibit the key biochemical hallmarks of ALD.

### 3.2 VLCFAs shift the lipid profile of ALD hiPSC‐derived astrocytes

**FIGURE 2:**
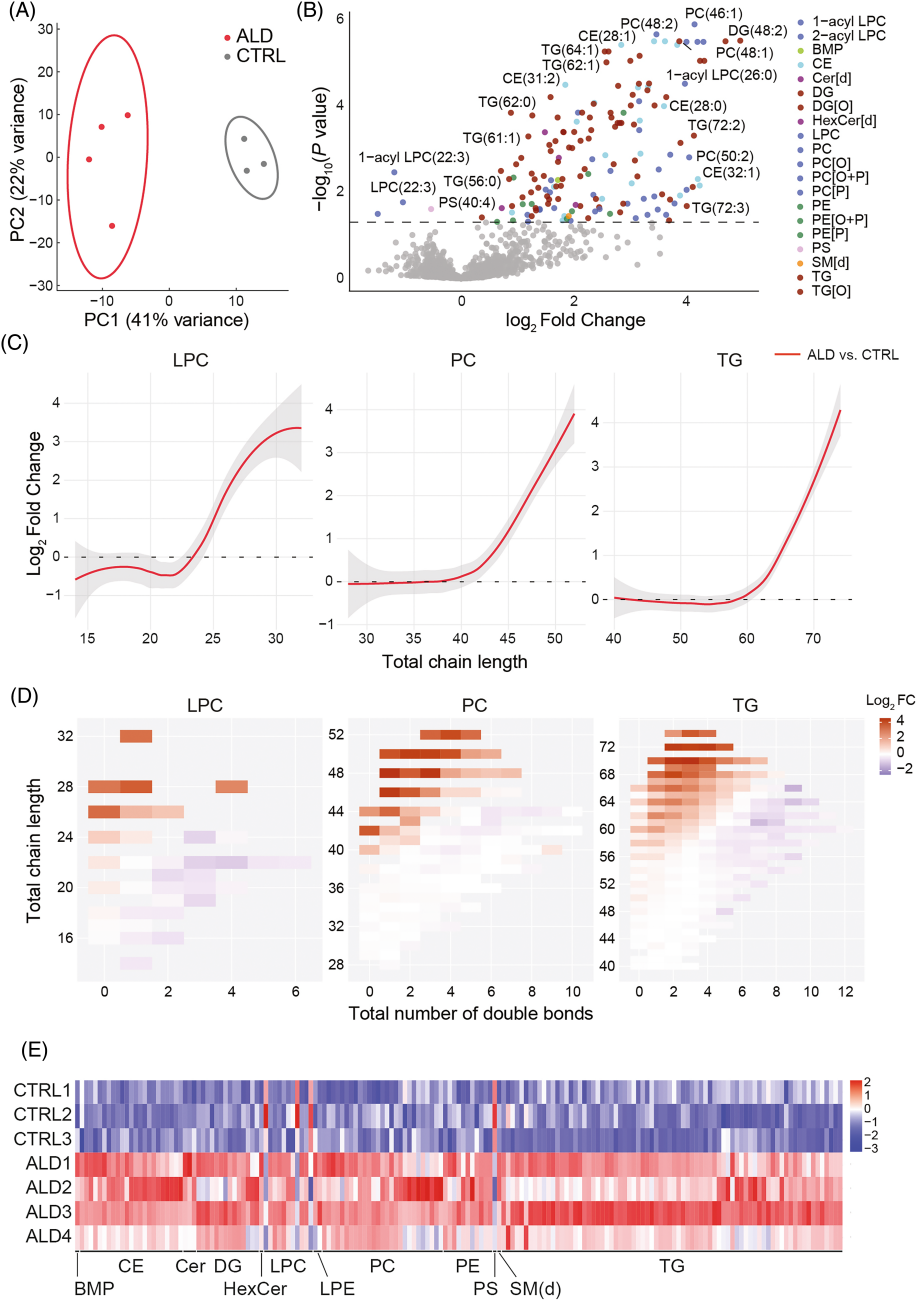
Differentially abundant lipids in adrenoleukodystrophy (ALD) human‐induced pluripotent stem cells (hiPSC)‐derived astrocytes. (A) Principal component analysis plot of lipid profile of control and ALD hiPSC‐derived astrocytes at Day 90. (B) Volcano plot comparing the lipid profiles of control and ALD hiPSC‐derived astrocytes at Day 90. The *x*‐axis shows the log fold change in ALD hiPSC‐derived astrocytes and the *y*‐axis shows the significance of this change expressed as –log (*p* value). Phospholipids are expressed as C(XX:Y), where XX is the total number of carbon atoms and Y is the total number of double bonds in the fatty acyl chains. This analysis, without fragmentation, does not allow specification of which fatty acid is at the *sn*‐1 or *sn*‐2 position. (C) Lipid chain trend plots of lysophosphatidylchloline (LPC) (left panel), phosphatidylcholine (PC) (middle panel), and triglycerides (TG) (right panel). The *x*‐axis represents the chain length of each lipid class, and the *y*‐axis represents the log2 fold change between CTRL and ALD hiPSC‐derived astrocytes. Note an increase in LPC, PC, and TG species containing fatty acids with a total number of more than 24, 44, and 64 carbon atoms, respectively, indicating an enrichment in very long‐chain fatty acid. (D) Saturation heat maps of LPC (left panel), PC (middle panel), and TG (right panel). The *x*‐axis represents chain unsaturation, and the y‐axis represents chain length. Plotted is the log2 fold change between CTRL and ALD hiPSC‐derived astrocytes. Group differences in the lipidomics data were tested with a linear mixed‐effects model (LMM), where subject (cell line)‐specific deviations from the group mean were modeled with random intercepts. Prior to analysis, the lipidomics data were normalized to the internal standards and then log transformed. (E) Heatmap of significantly differentially abundant lipids. Each row represents one hiPSC‐derived astrocyte cell line, and the *x*‐axis shows the different lipid classes. LPE, lysophosphatidylethanolamine; PE, phosphatidylethanolamine; PS, phosphatidylserine; SM, sphingomyelin.

Next, we investigated how deficits in VLCFA metabolism in ALD hiPSC‐derived astrocytes affect their lipid and metabolite profiles using untargeted lipidomics and metabolomics. Analysis of 90‐days‐old control and ALD hiPSC‐derived astrocyte pellets identified 1796 lipid species and 134 metabolites. Principal component analysis (PCA) revealed a clear separation between the lipidomes of control and ALD hiPSC‐derived astrocytes (Figure 2A). There was a skewed distribution of the significant hits toward an increase in numerous differentially abundant lipids in ALD hiPSC‐derived astrocytes compared to control hiPSC‐derived astrocytes. Consistent with the LC–MS/MS results, LPC(26:0) levels were increased (Figure 2B). In addition, PS(40:4), 2‐acyl LPC(22:4), 1‐acyl LPC(22:3), and LPC(22:3) were reduced in ALD astrocytes. In contrast, the metabolomes did not show major differences between control and ALD hiPSC‐derived astrocytes (Figure S3A,B).

To further characterize the lipid profile of ALD hiPSC‐derived astrocytes, we analyzed the total chain length of lipids containing one (LPC), two (PC), or three (TG) fatty acids. The resulting lipid chain trend plots revealed a profound increase in LPC, PC, and TG species containing more than 24, 44, and 64 carbon atoms, respectively, highlighting an enrichment of VLCFAs in these lipid classes (Figure 2C). In addition, the saturation heatmaps showed that lipid species with longer chain lengths and fewer double bonds exhibited a greater fold change between control and ALD (Figure 2D).

We investigated which lipid classes were mainly affected in the lipid profile of ALD hiPSC‐derived astrocytes. Among the 39 identified lipid classes, we observed significantly differentially abundant lipids in 12 major lipid classes (Figure 2E). TG showed the largest number of significantly differentially abundant lipid species (47), followed by alkyl diacylglycerols (TG(O)), CE, and PC (27, 22, and 20, respectively) (Figure S4A).

TG are found in lipid droplets together with other neutral lipids, mainly sterol esters such as CE.^35^ Analysis of the lipid droplet pool in control and ALD hiPSC‐derived astrocytes using the lipophilic neutral lipid dye Sudan III (Figure S4B) revealed no differences in lipid droplet abundance between control and ALD hiPSC‐derived astrocytes (Figure S4C,D).

Taken together, ALD hiPSC‐derived astrocytes have a relatively normal metabolite profile, but a profoundly altered lipid profile characterized by increased levels of VLCFA‐containing lipids.

### 3.3 Response of hiPSC‐derived astrocytes to VLCFA

**FIGURE 3:**
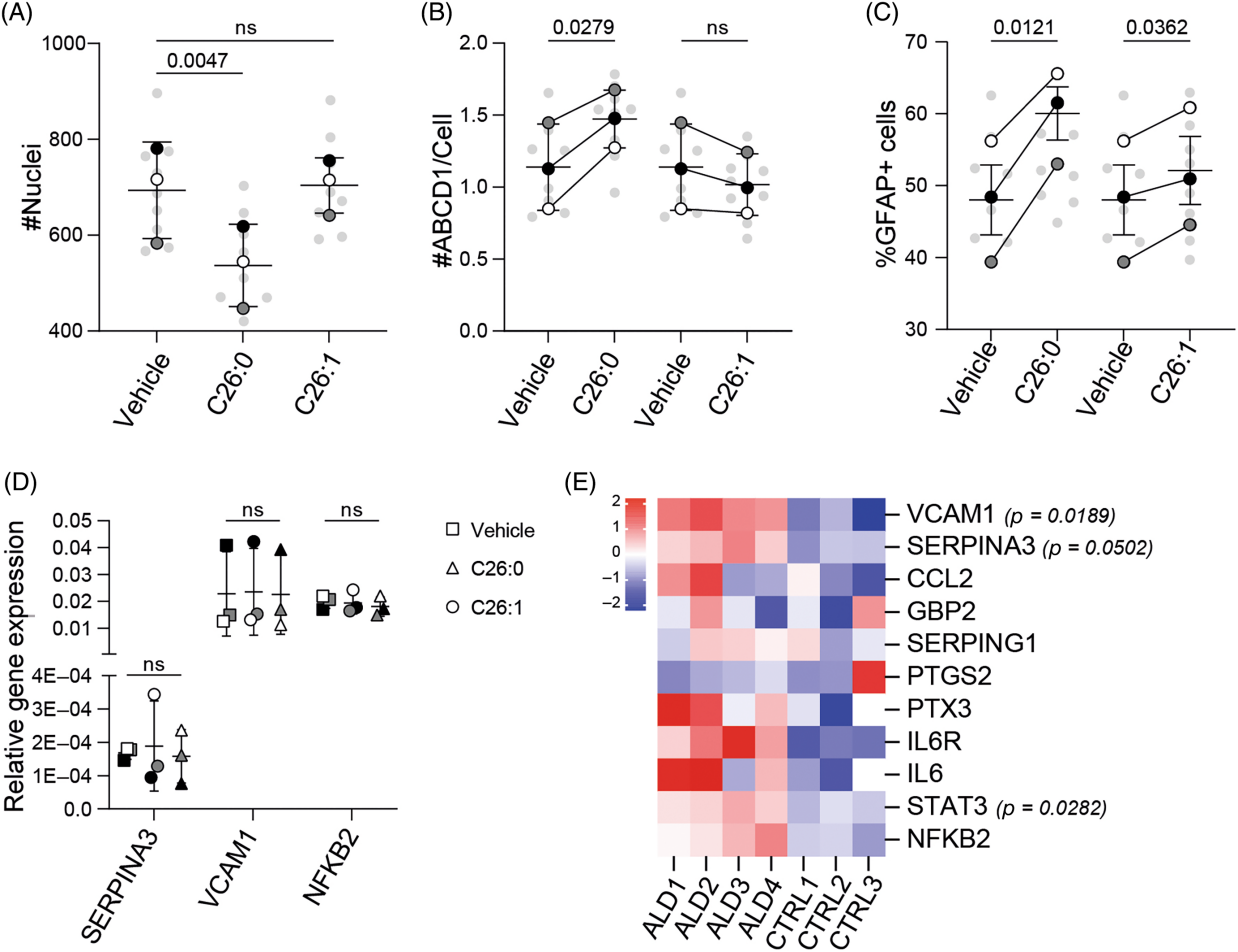
Response of human‐induced pluripotent stem cells (hiPSC)‐derived astrocytes to very long‐chain fatty acid. (A) Quantification of nuclei in control (*n* = 3) hiPSC‐derived astrocytes after 24 h treatment with 10 μM C26:0 or C26:1 at Day ~90; three independent batches per line; mean ± SD; (B) ABCD1 abundance in control (*n* = 3) hiPSC‐derived astrocytes after 24 h treatment with 10 μM C26:0 and C26:1 compared to vehicle condition at Day ~90; three independent batches per line; mean ± SD. (C) Quantification of %GFAP^+^ astrocytes in control (*n* = 3) hiPSC‐derived astrocytes after 24 h treatment with 10 μM C26:0 and C26:1 compared to vehicle condition (0.17% DMSO) at Day ~90; three independent batches per line; mean ± SD. In (A–C), the mean values of each donor are color coded (black, dark gray, and white) and the mean of 12 fields within three 96‐well plates of each differentiation batch (*n* = 3) is shown in gray in the background. *p* values were calculated by paired *t*‐test with the mean of 12 fields within three 96 well‐plate wells of each differentiation batch nested per group (control *n* = 3 and adrenoleukodystrophy [ALD] *n* = 4). (D) SERPINA3, VCAM1, and NFκB2 relative gene expression levels in control hiPSC‐derived astrocytes (*n* = 3) after 24 h treatment with 10 μM C26:0 or C26:1 compared to vehicle (0.17% DMSO) condition at Day ~90; mean ± SD. *p* values were calculated by paired *t*‐test with the mean of 2 − ∆∆Ct values of each differentiation batch, previously normalized against the housekeeping gene (control *n* = 3); three independent batches per line. (E) Heatmap of response‐associated genes in hiPSC‐derived astrocytes; mean fold changes of three independent batches per line against the control group mean for ALD lines and against the ALD group mean for control lines. *p* values were calculated by unpaired *t*‐test with Welch’ correction with the mean of 2 − ∆∆Ct values of each differentiation batch, previously normalized against the housekeeping gene (control *n* = 3 and ALD *n* = 4) and are shown as numbers when significant; three independent batches per line.

To better understand how hiPSC‐derived astrocytes respond to VLCFA, we incubated control cultures with C26:0 and C26:1. We observed a decrease in cell number when cultures were incubated with saturated VLCFA, whereas no such effect was observed when cultures were incubated with monounsaturated VLCFA (Figure 3A). In addition, we observed an increase in ABCD1 abundance in control hiPSC‐derived astrocytes incubated with C26:0, whereas hiPSC‐derived astrocytes incubated with C26:1 showed a trend suggesting a decrease (*p* = 0.1373, Figure 3B). These results suggest that saturated, but not monounsaturated, VLCFA may induce a response that stimulates VLCFA degradation in hiPSC‐derived astrocytes.

We next investigated whether the accumulation of VLCFA could induce astrocyte reactivity that could trigger deficits in the neuronal microenvironment, as previously shown.^36^ Acute exposure of control hiPSC‐derived astrocytes to VLCFA increased the percentage of GFAP‐positive astrocytes (Figure 3C). GFAP is not expressed in all astrocytes, and an increase in its levels alone is no longer considered a definitive marker of astrocyte reactivity by current standards.^37^, ^38^ Therefore, we evaluated the levels of pan‐reactive‐associated genes in VLCFA‐treated astrocytes and no differences were observed (Figure 3D). Next, we evaluated the expression levels of a selection of reactive astrocyte markers in untreated ALD hiPSC‐derived astrocytes^36^, ^39^ and observed an upregulation of reactive astrocyte‐associated genes (Figure 3E). These results suggest that hiPSC‐derived astrocytes respond to acute exposure to VLCFA, but this does not lead to astrocyte reactivity. In contrast, ALD hiPSC‐derived astrocytes that chronically accumulate VLCFA could acquire a reactive phenotype.

### 3.4 ALD hiPSC‐derived astrocytes fail to support neurons in co‐cultures

**FIGURE 4:**
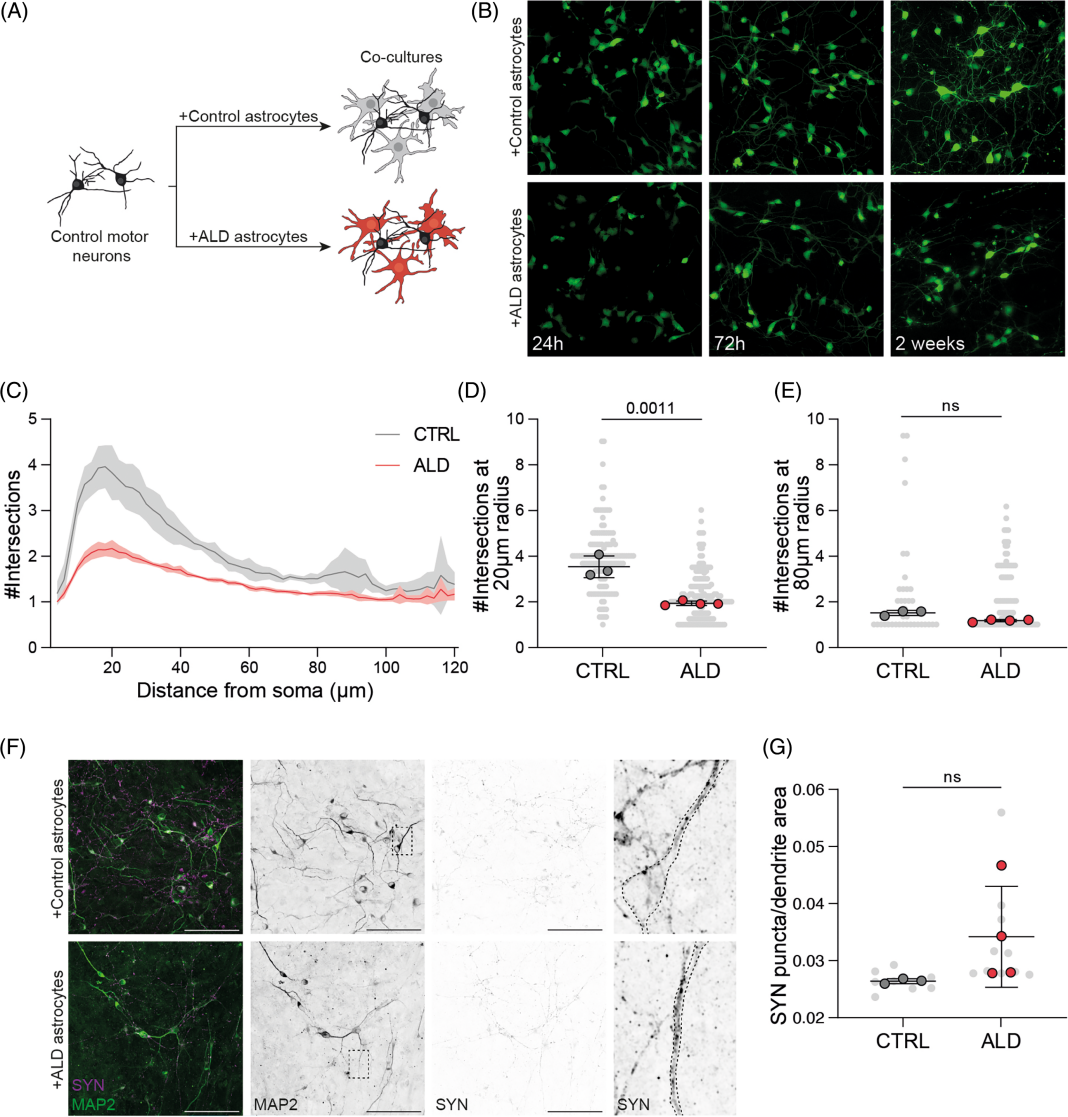
Adrenoleukodystrophy (ALD) human‐induced pluripotent stem cells (hiPSC)‐derived astrocytes fail to support motor neurons in vitro. (A) Schematic illustrating the co‐culture approach of control hiPSC‐derived motor neurons co‐cultured with either control (gray) or ALD (red) hiPSC‐derived astrocytes. (B) Cropped live‐cell images of GFP^+^ motor neurons co‐cultured with control (upper panel) and ALD (lower panel) hiPSC‐derived astrocytes at 24 h, 72 h, and 2 weeks. (C) Sholl intersection profile of MAP2 dendritic arborization of control hiPSC‐derived motor neurons co‐cultured with control hiPSC‐derived astrocytes (gray line; *n* = 3) and ALD hiPSC‐derived astrocytes (red line; *n* = 4). Three independent batches per astrocyte line; two to three coverslips per line; 45 ± 23 neurons/coverslip; mean ± SD. (D, E) Stratified analyses were also performed on two radius ranges, 20–29 and 80–89 μm. (F) Cropped confocal images of control hiPSC‐derived motor neurons immunostained for MAP2 and SYNAPTOPHYSIN (SYN) co‐cultured with control hiPSC‐derived astrocytes (top panel) and ALD hiPSC‐derived astrocytes (bottom panel). (G) Quantification of SYN puncta (total number of vesicles divided by the dendrite length) within the dendritic mask defined by MAP2 positivity. Three independent batches per astrocyte line; two to three coverslips per line; mean ± SD. Mean values are shown in color and the mean of four fields from each differentiation batch is shown in gray in the background. *p* values were calculated by two‐tailed nested *t*‐test with the mean of four fields of each differentiation batch nested per group (control *n* = 3 and ALD *n* = 4) and are shown as numbers when significant.

Next, we examined the functional effects of ALD hiPSC‐derived astrocytes on the neuron–glia microenvironment by co‐culturing control hiPSC‐derived motor neurons with either control or ALD hiPSC‐derived astrocytes (Figure 4A). We first generated control hiPSC‐derived motor neuron progenitors that were positive for OLIG2 and NKX6.1 at differentiation Day 13 (Figure S5B), which, when differentiated into neurons (Figure S5D), showed positivity for NeuN, SYN, ISL1, and HB9 at differentiation Day 38 (Figure S5E,F). In control co‐cultures, control hiPSC motor neurons presented ABCD1 (Figure S5G,H). At Day 22 of differentiation, control hiPSC‐derived motor neuron progenitors were plated on either control or ALD hiPSC‐derived astrocytes (Figure 4A). In parallel, we generated co‐cultures with control motor neurons from a human pluripotent stem cell line with stable expression of GFP, which were plated on control and ALD hiPSC‐derived astrocytes to monitor neuronal morphology in real time. Live cell imaging of the co‐cultures showed that the cells had a neuronal progenitor morphology 24 h after plating (Figure 4B). After 3 days, the plated progenitors developed a young neuron‐like morphology with extending neurites. After 2 weeks, hiPSC‐derived motor neurons developed a more complex morphology when plated with control hiPSC‐derived astrocytes but maintained a simpler morphology when plated with ALD hiPSC‐derived astrocytes (Figure 4B). To measure neuronal complexity, we fixed the co‐cultures after 2 weeks and assessed dendritic arborization based on MAP2 reconstructions. Sholl analysis revealed that hiPSC‐derived motor neurons cultured with ALD hiPSC‐derived astrocytes showed reduced morphological complexity compared to those cultured with control hiPSC‐derived astrocytes, with an average of 1.32‐fold fewer intersections (Figure 4C). The overall difference in log‐transformed intersections between ALD and control was evaluated using a linear mixed effects model (*β*_log2(*x* + 1)_ = 0.40, SE = 0.053, *T*‐value = 7.38, *p* = 7.0 × 10^−4^). Neurons co‐cultured with ALD hiPSC‐derived astrocytes showed a decrease in the number of intersections near the soma (20 μm radius, Figure 4D), whereas the number of intersections further away from the soma showed no differences (80 μm radius; Figure 4E). Within the 20–29 μm range, neurons from the ALD condition had on average 1.22 fewer intersections compared to the control condition (*β*_log2(*x* + 1)_ = 0.61, SE = 0.09, *T*‐value = 6.74, *p* = 1.1 × 10^−3^), and no difference was seen within the 80–89 μm range (*β*_log2(*x* + 1)_ = 0.07, SE = 0.038, *T*‐value = 1.90, *p* = 0.12).

Astrocytic trophic support is essential for synaptogenesis.^40^ Therefore, we analyzed the number of presynaptic vesicles by quantifying SYN puncta per MAP2‐positive area of control hiPSC‐derived motor neurons co‐cultured with control and ALD hiPSC‐derived astrocytes (Figure 4F,G). Although it did not reach statistical significance, on average we observed an increase in presynaptic SYN puncta in the co‐cultures with ALD hiPSC‐derived astrocytes.

These results suggest that ALD hiPSC‐derived astrocytes have reduced neuronal support in vitro.

## 4 DISCUSSION

Post‐mortem studies have clearly defined the structures affected in ALD, but this represents late‐stage disease characterized by axonal loss in the spinal cord and destruction and glial scarring of the cerebral white matter.^41^, ^42^ It is unclear which cell types are initially involved and drive the pathology. In this study, we aimed to gain more insight into the role of astrocytes in ALD disease manifestation using hiPSC technology. To validate ALD hiPSC‐derived astrocytes as a cellular model for ALD, we confirmed that ALD hiPSC‐derived astrocytes recapitulate the biochemical deficits identified in patients, including reduced peroxisomal VLCFA β‐oxidation and VLCFA accumulation. In addition, untargeted lipidomics revealed a marked shift in the lipid profile of ALD hiPSC‐derived astrocytes. Furthermore, ALD hiPSC‐derived astrocytes exhibited a reactive‐associated phenotype that was not induced by acute VLCFA exposure in control hiPSC‐derived astrocytes. Since ALD hiPSC‐derived astrocytes failed to support neuronal maturation, a role for astrocytes in central nervous system pathology is further confirmed. In conclusion, we report that astrocytes are profoundly affected by an ALD genetic background and subsequent biochemical profile, highlighting their potential involvement in ALD pathophysiology.

In this study, we performed a comprehensive characterization of the lipid profile of ALD hiPSC‐derived astrocytes. Our findings are consistent with a characteristic ALD lipid signature^43^, ^44^ and confirm the accumulation of VLCFA in ALD hiPSC‐derived astrocytes as previously reported.^45^ Importantly, we extend these findings by demonstrating the incorporation of VLCFAs into complex lipids, providing new insights into the lipid changes associated with ALD. Our metabolomics assay did not reveal significant differences between ALD and control astrocytes, likely due to its focus on pathways not central to lipid dysfunction in ALD, including amino acid metabolism, glycolysis, purine and pyrimidine metabolism, and the TCA cycle. We show a profound increase in VLCFA‐containing TG and CE in ALD hiPSC‐derived astrocytes. TG and CE play a central role in energy storage and are localized in intracellular lipid droplets.^35^ The accumulation of lipid droplets is associated with astrocyte stress, and is increasingly recognized as an underlying factor in neurodegeneration.^46^, ^47^ However, we found no differences in lipid droplet abundance in ALD hiPSC‐derived astrocytes. The finding that TG and CE are enriched in VLCFA suggests a potential protective cellular storage mechanism to cope with VLCFA overabundance by altering lipid droplet composition. However, hiPSC‐derived astrocytes challenged with long‐chain monounsaturated fatty acids secrete TG‐rich lipid particles in an APOE‐dependent process.^48^ Consistent with a reduced ability to degrade FAs,^49^ *APOE4* hiPSC‐derived astrocytes accumulated unsaturated TGs, had increased lipid droplet abundance, and exhibited the highest TG secretion among the different APOE isoforms.^48^, ^50^ Relevantly, *APOE4* single nucleotide polymorphism (SNP) variants have been associated with increased clinical cerebral disease severity in cerebral ALD cases.^51^ In the context of ALD, the secretion of VLCFA‐TG lipid particles from ALD astrocytes would be detrimental to the neuronal milieu, possibly acting as toxic particles. Therefore, the role of astrocyte‐derived VLCFA‐TG may be bidirectional, with both neuroprotective and toxic properties. Further study of lipid droplet secretion and composition is likely to provide new insights into the contribution of VLCFA‐TG in the neuropathogenesis and neurodegeneration of ALD.

It is important to note that while our ALD hiPSC‐derived astrocyte model system was able to capture key biochemical hallmarks of ALD and expand our knowledge of cell‐autonomous ALD hiPSC‐derived astrocyte alterations, the impact of the ALD genetic background on other disease‐relevant cell types and their metabolic communication still requires further investigation. Crosstalk between neurons and glia in the regulation of lipid metabolism is critical for the maintenance of brain homeostasis.^21^, ^25^ In support of this, we found that control hiPSC‐derived motor neurons developed a complex dendritic arborization when co‐cultured with control astrocytes but developed a less complex morphology when co‐cultured with ALD hiPSC‐derived astrocytes. Importantly, the decrease in the number of intersections near the soma suggests that the primary dendritic tree of neurons co‐cultured with ALD hiPSC‐derived astrocytes was specifically compromised. This finding highlights the importance of the microenvironment in promoting neuronal health. In the context of ALD, where ABCD1 is predominantly present in astrocytes and microglia,^10^ ALD‐specific lipid dyshomeostasis in both cell types likely results in a combined effect on VLCFA accumulation within the central nervous system, thereby increasing the vulnerability of neurons to VLCFA toxicity. Therefore, future research should not only focus on single cell types, but also explore the communication between different disease‐relevant cell types.

VLCFA‐containing lipids have been implicated in the pathophysiology of ALD. High levels of VLCFA have been associated with oxidative stress, mitochondrial dysfunction, increased endoplasmic reticulum (ER) stress, and membrane dysfunction^44^, ^52^, ^53^, ^54^, ^55^ In addition, lipid profiling of postmortem cerebral ALD brains revealed a significant increase in VLCFA‐containing CE in active demyelinating areas and VLCFA‐containing PC in both affected and intact white matter.^56^ Furthermore, VLCFA‐enriched LPC are cytotoxic, as intracortical injection of LPC(24:0) induced widespread microglial activation and apoptosis in wild‐type mice.^57^ Incorporation of excessive VLCFA into PC could lead to disruption of membrane integrity, which in turn could affect neuronal signaling, myelin structure, and overall membrane fluidity. This is in contrast to the essential role of VLCFA in neuronal differentiation and development, where their synthesis plays a pivotal role in the formation of lipid rafts necessary for neuronal polarity.^58^ This highlights the need to maintain a delicate balance of VLCFA levels for healthy brain function.

In response to disease and injury, astrocytes can undergo morphological, transcriptional, and functional changes and transition into a “reactive” state with potential neuroprotective and neurotoxic effects.^37^ Recently, Mi et al.^22^ reported that impaired astrocytic mitochondrial fatty acid metabolism is associated with astrocyte reactivity.^22^ In addition, incubation of wild‐type mouse astrocytes with exogenous oleate (C18:1) also resulted in astrocyte reactivity.^22^ Here, we observed that impaired astrocytic peroxisomal VLCFA metabolism in ALD hiPSC‐derived astrocytes is accompanied by a reactive‐associated astrocyte profile. However, oversupply of C26:0 or C26:1 to control astrocytes did not translate into astrocyte reactivity. Importantly, analysis of the lipid profile of cytokine‐induced reactive astrocytes revealed that saturated lipids, primarily saturated LCFA and VLCFA, were elevated and drove neurotoxicity.^59^ Taken together, fatty acid metabolic disease models appear to be related to astrocyte reactivity, and reactive astrocytes exhibit changes in their lipid profile, and while long‐chain monounsaturated fatty acids (C18:1) induce astrocyte reactivity, saturated LCFA and VLCFA may primarily mediate toxicity. Importantly, chain length and saturation state‐specific effects have also been demonstrated in human primary fibroblasts. Exposure of ALD fibroblasts to saturated LCFA, monounsaturated LCFA, or monounsaturated VLCFA did not result in cellular stress. Only exposure of ALD cells to saturated VLCFA (C24:0 and C26:0) caused ER stress.^52^

## 5 CONCLUSION

This study confirms that ALD hiPSC‐derived astrocytes are a suitable cellular in vitro model system to study the cellular impact of the ALD genetic background on astrocytes. We report profound changes in the lipid profile and phenotype of ALD hiPSC‐derived astrocytes, suggesting that astrocytes may play an important role in the pathophysiology of ALD. Future research focusing on how the cell‐autonomous changes observed in ALD hiPSC‐derived astrocytes affect the brain microenvironment and the different cell types present is likely to provide valuable insights into our understanding of ALD pathogenesis.

## AUTHOR CONTRIBUTIONS

RMF, ME, SK, and VMH conceived the project. ME, SK, and VMH obtained funding. RMF, YRJJ, IMED, and NB performed the laboratory analyses. RMF, YRJJ, J‐BvK, and CR analyzed the data. SK and VMH provided the resources for the project. ME, SK, and VMH supervised the project. RMF, YRJJ, ME, SK, and VMH wrote the manuscript.

## FUNDING INFORMATION

This study was financially supported by grants from the European Leukodystrophy Association (ELA International), grant number: 2019‐020C2 (to Stephan Kemp and Vivi M. Heine), Stichting Metakids, grant number: 2023‐102 (to Stephan Kemp and Vivi M. Heine), the Netherlands Organization for Scientific Research (NWO) (VIDI 016.196.310) (to Marc Engelen), and Amsterdam UMC (FlexOIO) (to Marc Engelen).

## CONFLICT OF INTEREST STATEMENT

Roberto Montoro Ferrer, Yorrick Jaspers, Inge Dijkstra, Nicole Breeuwsma, Jan‐Bert van Klinken, Cato Romero, and Vivi Heine declare that they have no conflict of interest. Marc Engelen participates in the advisory board the United Leukodystrophy Foundation (unpaid); Stephan Kemp participates in the advisory boards for ALD Connect (unpaid), the European Leukodystrophy Association (unpaid), Alex, The Leukodystrophy Charity (unpaid), and the United Leukodystrophy Foundation (unpaid).

## ETHICS STATEMENT

This article does not contain any studies with human subjects performed by any of the authors.

## PATIENT CONSENT

This article does not contain any studies with human subjects performed by any of the authors.

## ANIMAL RIGHTS

This article does not contain any studies with animal subjects performed by any of the authors.

## Supporting information


**Data S1.** Supporting Information.

## Data Availability

The data that support the findings of this study are available on request from the corresponding author.
