# Supplementary material for: Altered lipid profile and reduced neuronal support in human induced pluripotent stem cell‐derived astrocytes from adrenoleukodystrophy patients
Source: J Inherit Metab Dis. 2024 Dec 20;48(1):e12832. doi: 10.1002/jimd.12832 (PMC11660744; doi:10.1002/jimd.12832)
Supplement: Supplementary file 1 — Data S1. Supporting Information. [file JIMD-48-0-s001.pdf]

Supplemental Figure 1: hiPSC characterization

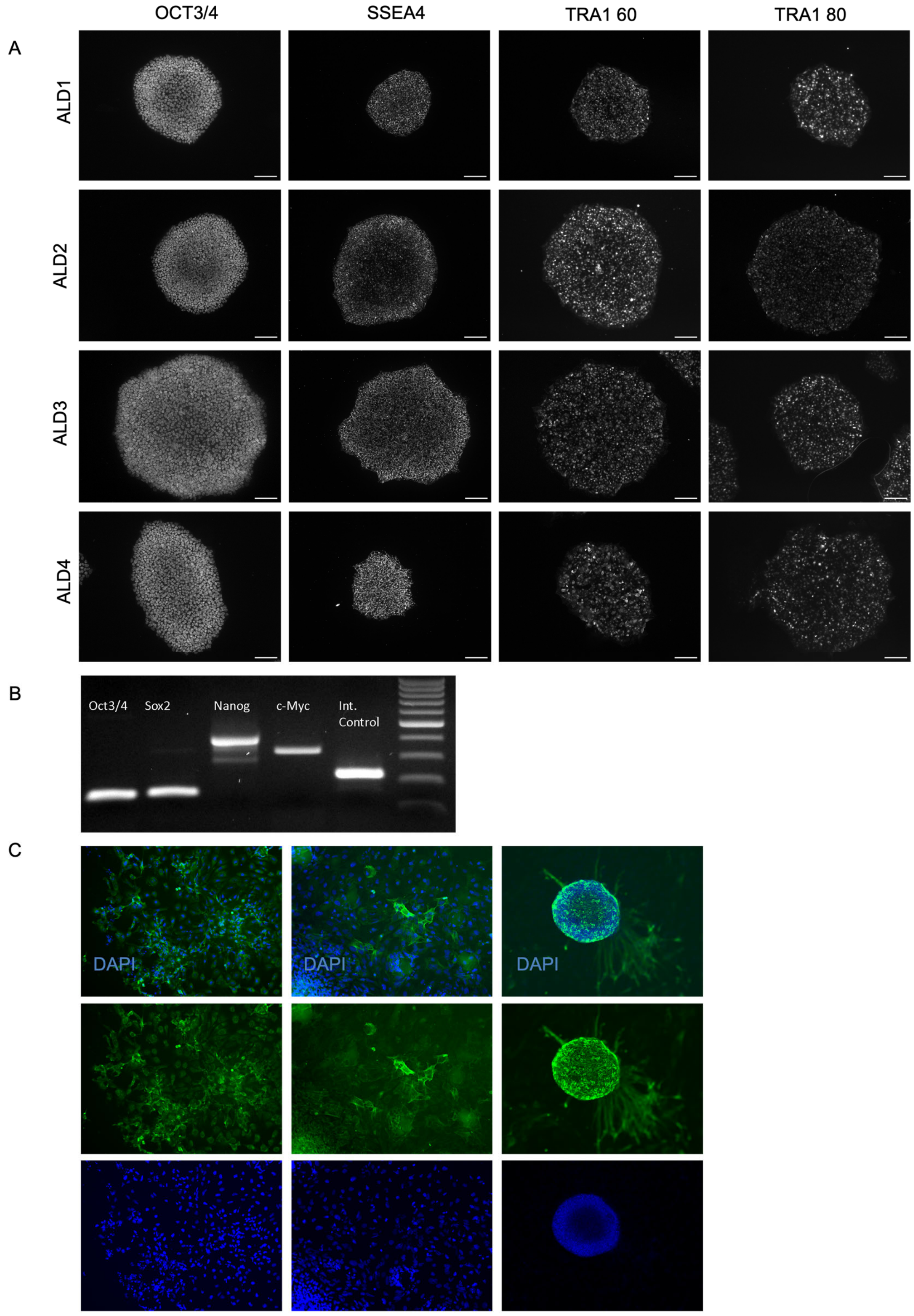

A. Representative immunofluorescence images of hiPSC colonies from all ALD lines stained for the pluripotency markers OCT3/4, SSEA4, TRA1-60, and TRA1-80.

B. A representative RT-PCR showing expression of the pluripotency markers *OCT3/4*, *SOX2*, *NANOG*, and *C-MYC*.

C. Representative immunofluorescence images of all three germ layer markers AFP, α-SMA, and ß-III-TUBULIN.

Supplemental Figure 2: hiPSC-derived astrocytes characterization

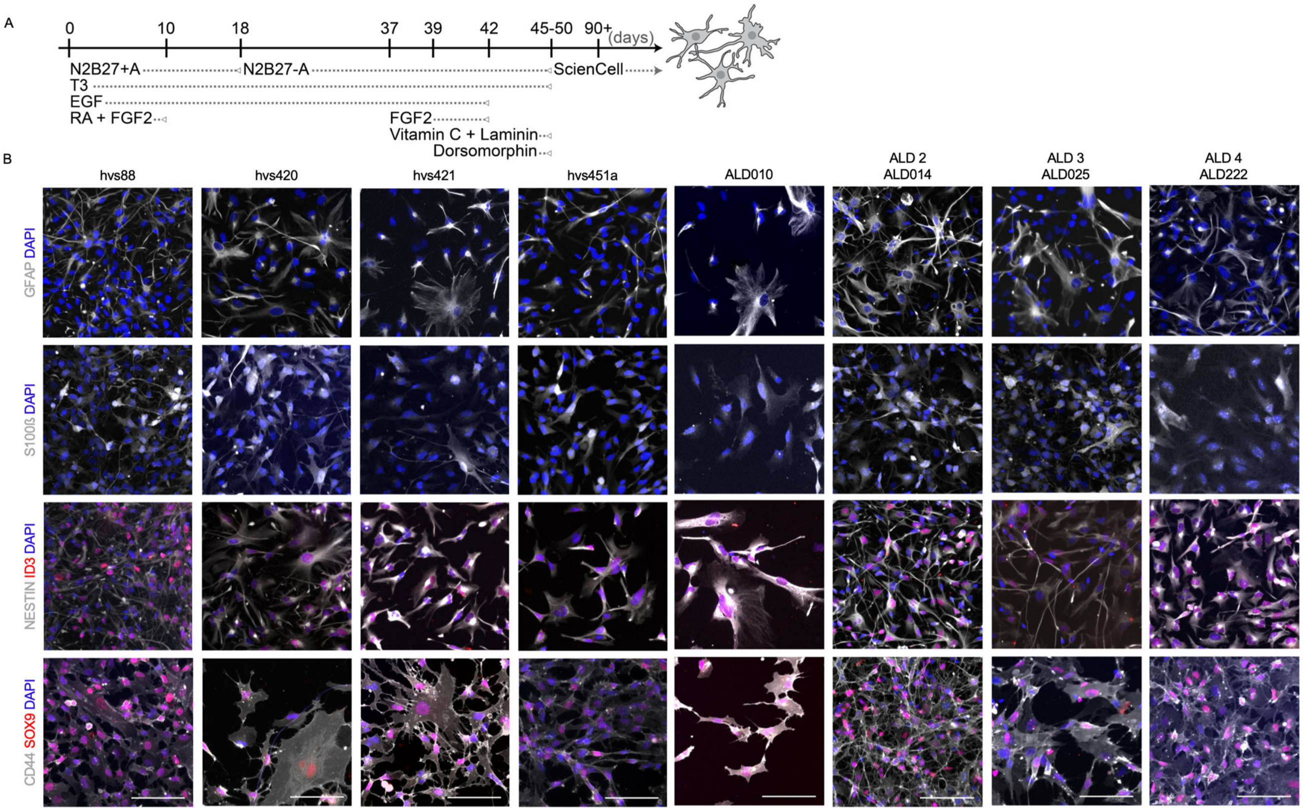

A. Schematic illustrating the main stages of the hiPSC-derived astrocyte differentiation protocol.

B. Immunofluorescence images of control and ALD hiPSC-derived astrocytes positive for the astrocyte-associated markers GFAP, S100ß, ID3, NESTIN, CD44 and SOX9 at day ~60 (scale bar, 100 µm).

Supplemental Figure 3: Metabolomics analysis

A

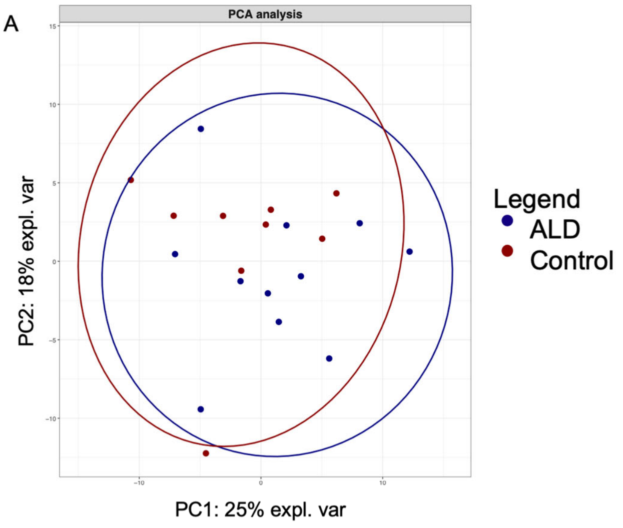

B

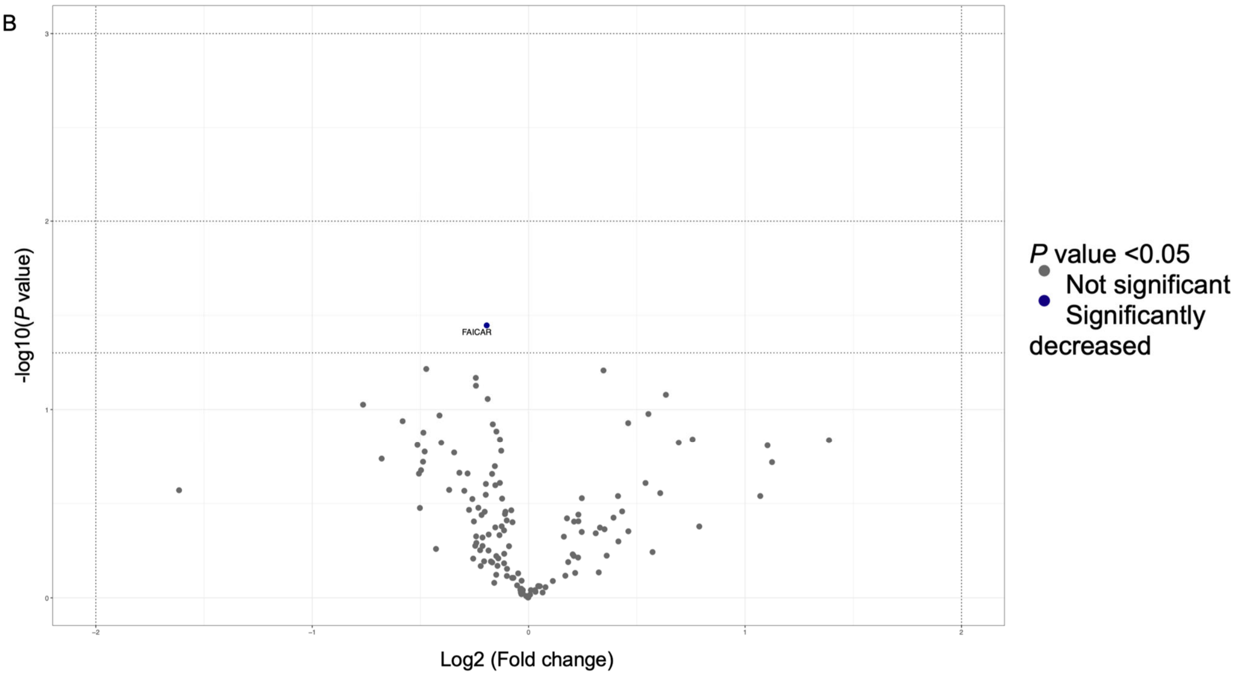

A. Metabolite profile PCA plot of control and ALD hiPSC-derived astrocytes at day 90.

B. Volcano plot comparing the metabolic profiles of control and ALD hiPSC-derived astrocytes at day 90. The x-axis shows the log fold change in ALD hiPSC-derived astrocytes, and the y-axis shows the significance of this change expressed as –log (*P* value).

For the analysis of metabolomics data, group differences were assessed using a Welch's t-test.

Supplemental Figure 4: Alterations in lipid droplet integral lipids in ALD hiPSC-derived astrocytes

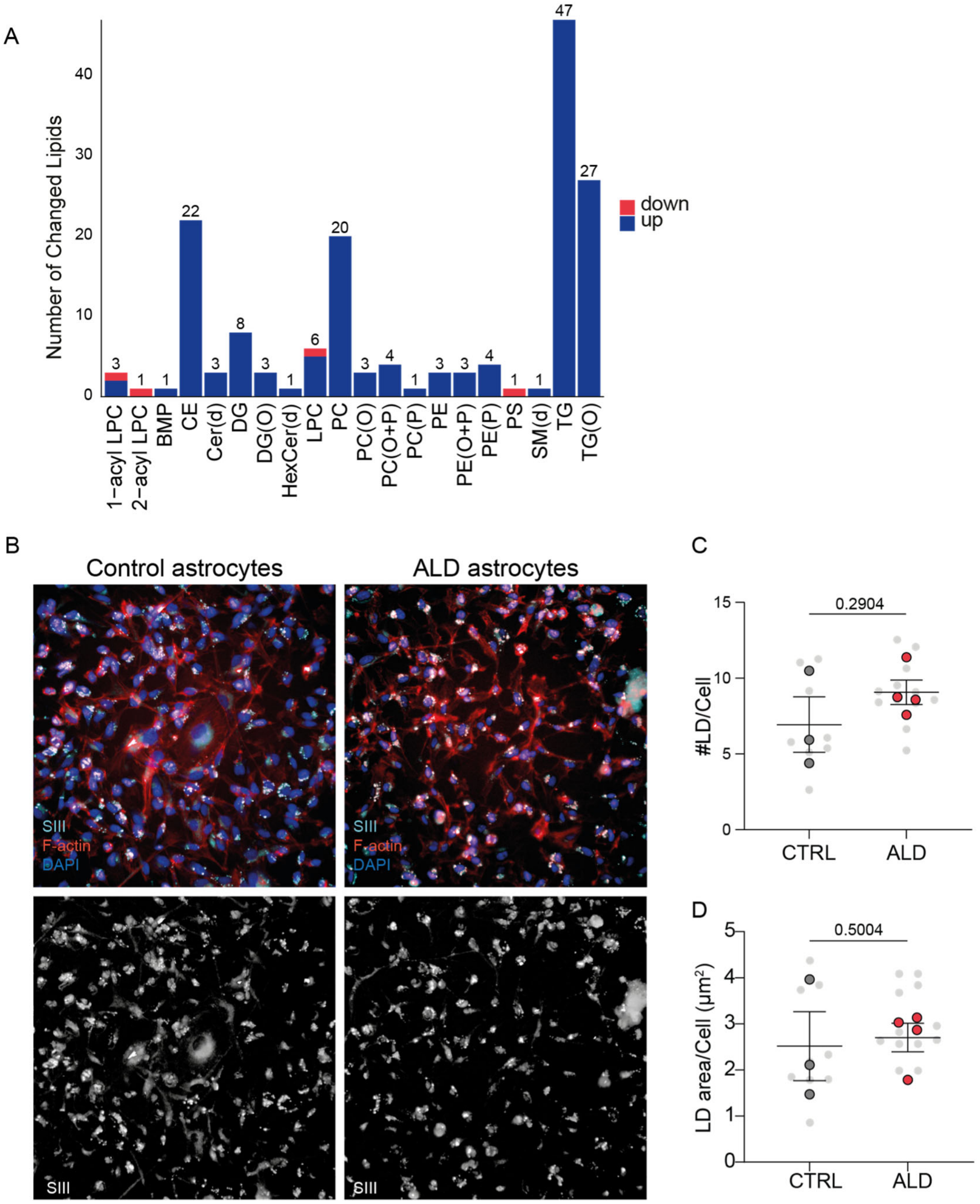

A. Quantification of significantly altered lipid species per lipid class. The total number of altered lipids per class is indicated within each bar.

B. Immunofluorescence image of control and ALD hiPSC-derived astrocytes immunostained for F-actin and stained with Sudan III to visualize lipid droplets (LDs); single channel images of LDs are shown in grey scale.

C. LD abundance per cell in control (n = 3) and ALD (n = 4) hiPSC-derived astrocytes at day ~90; 3 independent batches per line; mean ± SD; mean values of each donor are coloured and the mean of 12 fields within two wells from a 96-well plate of each differentiation batch (n = 3) are plotted in grey in the background. *P* values were calculated by two-tailed nested t-test with the mean of 12 fields two wells from a 96-well plate of each differentiation batch nested per group (control n = 3 and ALD n = 4).

D. LD area per cell in control (n = 3) and ALD (n = 4) hiPSC-derived astrocytes at day ~90; 3 independent batches per line; mean ± SD; mean values of each donor are coloured and the mean of 12 fields two wells from a 96-well plate of each differentiation batch (n = 3) are shown in grey in the background. *P* values were calculated by two-tailed nested t-test with the mean of 12 fields within two wells from a 96-well plate of each differentiation batch nested per group (control n = 3 and ALD n = 4).

Supplemental Figure 5: hiPSC-derived motor neuron and co-cultures characterization

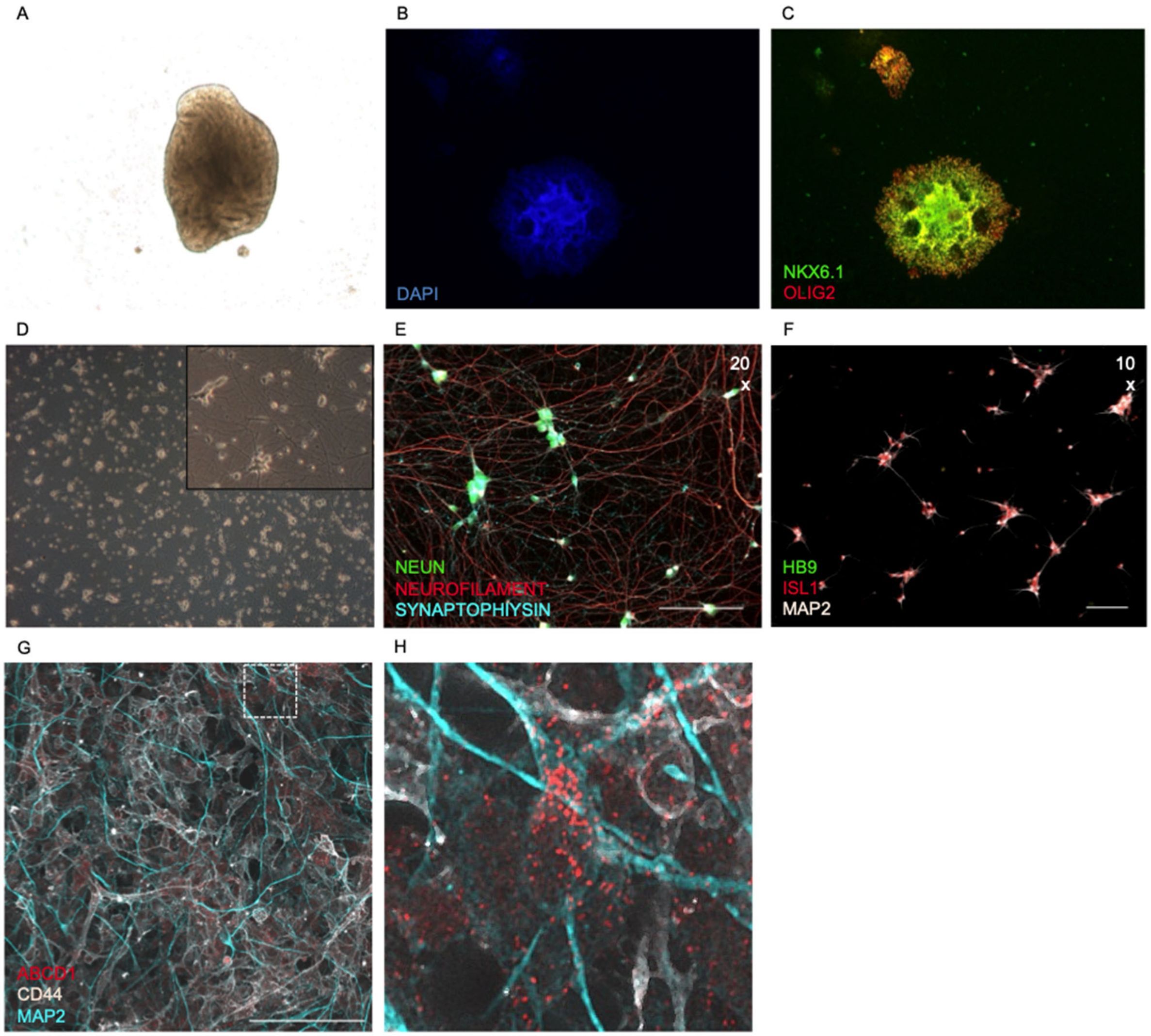

A. Bright field image of day 7 motor neuron differentiating embryoid bodies.

B, C. Immunofluorescence images of control day 13 plated hiPSC-derived motor neuron progenitor EBs against markers NKX6.1 and OLIG2.

D. Bright field image of hiPSC-derived motor neurons.

E, F. Immunofluorescence images of control hiPSC-derived motor neurons against NeuN, NEUROFILAMENT, SYNAPTOPHYSIN, HB9, and ISL1 (scale bar, 100μm).

G. Immunofluorescence image of 2 weeks co-culture of control hiPSC-derived astrocytes (CD44) and control hiPSC-derived motor neurons (MAP2) positive for ABCD1 (scale bar, 100μm).

H. A magnified image of the boxed area indicated in G showing a MAP2 neuron with ABCD1 puncta.

Supplementary Table 1. hiPSC lines

Cell line	Sex	Age	Phenotype	Mutation	ABCD1 presence (IF)	Reprogramming method	Comments
Control 1 (hvs88)	Male	74 days	Control	No	NA	Lentivirus	
Control 2 (hvs420)	Male	21 years	Control	No	NA	Lentivirus	
Control 3 (hvs421 (1)/ hvs451 (2))	Male	19 years	Control	No	NA	Lentivirus (1)/ Sendai virus (2)	Same donor line, different clones
WA09 Cre-Lox GFP Control 4	Female	NA	Control	No	NA	NA	Not used in quantifications
ALD1 (ALD010)	Male	34 years	Cerebral ALD	c.1390C>T (p.Arg464*)	Absent	Lentivirus	
ALD2 (ALD014)	Male	76 years	Cerebral ALD	c.659T>C (p.Leu220Pro)	Reduced	Lentivirus	
ALD3 (ALD025)	Male	21 years	Cerebral ALD	c.1866-10G>A (p.Pro623fs*?)	Absent	Lentivirus	
ALD4 (ALD222)	Male	27 years	Spinal cord disease	c.446G>A (p.Ser149Asn)	Present	Lentivirus	

Supplementary Table 2. Primer list

PRIMER NAME	SEQUENCE
VCAM F	AACACTTTATGTCAATGTTGCCCC
VCAM R	CACAGGATTTTCGGAGCAGGA
SERPINA3 F	ACTCCAGACAGACGGCTTTG
SERPINA3 R	GTGAGCAGATCTCATCCTGCC
CCL2 F	TCAAACTGAAGCTCGCACTCT
CCL2 R	GGCATTGATTGCATCTGGC
GBP2 F	GGCCATGGACCAACTTCACTAT
GBP2 R	CAGCAGTGATGGGTTCTCCA
SERPING1 F	TCTCCTAACACTACCCCGCA
SERPING1 R	CAGCCCACACAGGTTAAGGT
PTGS2 F	CTCCCTTGGGTGTCAAAGGTAAA
PTGS2 R	AACTGATGCGTGAAGTGCTG
PTX3 F	GACTCCATCCCACTGAGGAC
PTX3 R	CAGCATGCGCTCTCTCATCT
IL6R F	ACATTCACAACATGGATGGTCAAG
IL6R R	AACTCCTCCTGGGCACGAA
IL6 F	CACTGGTCTTTTGGAGTTTGAG
IL6 R	GGACTTTTGTACTCATCTGCAC
STAT3 F	GCCGGAGAAACAGTTGGGAC
STAT3 R	TCTCAATCCAAGGGGCCAGA
NF-KB2 F	GGCAGACCAGTGTCATTGAGCA
NF-KB2 R	CAGCAGAAAGCTCACCACACTC
